# Intraspinal Plasticity Associated With the Development of Autonomic Dysreflexia After Complete Spinal Cord Injury

**DOI:** 10.3389/fncel.2019.00505

**Published:** 2019-11-08

**Authors:** Felicia M. Michael, Samir P. Patel, Alexander G. Rabchevsky

**Affiliations:** Department of Physiology, Spinal Cord and Brain Injury Research Center, University of Kentucky, Lexington, KY, United States

**Keywords:** propriospinal, interneuron, sympathetic preganglionic neurons, cardiovascular dysfunction, hypertension

## Abstract

Traumatic spinal cord injury (SCI) leads to disruption of sensory, motor and autonomic function, and triggers structural, physiological and biochemical changes that cause reorganization of existing circuits that affect functional recovery. Propriospinal neurons (PN) appear to be very plastic within the inhibitory microenvironment of the injured spinal cord by forming compensatory circuits that aid in relaying information across the lesion site and, thus, are being investigated for their potential to promote locomotor recovery after experimental SCI. Yet the role of PN plasticity in autonomic dysfunction is not well characterized, notably, the disruption of supraspinal modulatory signals to spinal sympathetic neurons after SCI at the sixth thoracic spinal segment or above resulting in autonomic dysreflexia (AD). This condition is characterized by unmodulated sympathetic reflexes triggering sporadic hypertension associated with baroreflex mediated bradycardia in response to noxious yet unperceived stimuli below the injury to reduce blood pressure. AD is frequently triggered by pelvic visceral distension (bowel and bladder), and there are documented structural relationships between injury-induced sprouting of pelvic visceral afferent C-fibers. Their excitation of lumbosacral PN, in turn, sprout and relay noxious visceral sensory stimuli to rostral disinhibited thoracic sympathetic preganglionic neurons (SPN) that manifest hypertension. Herein, we review evidence for maladaptive plasticity of PN in neural circuits mediating heightened sympathetic reflexes after complete high thoracic SCI that manifest cardiovascular dysfunction, as well as contemporary research methodologies being employed to unveil the precise contribution of PN plasticity to the pathophysiology underlying AD development.

## Introduction

Propriospinal neurons (PN) have intraspinal origins and project to interneurons in other spinal cord segments as reported in electrophysiological and tract-tracing studies in feline and rodent models (Alstermark et al., 1981; Chung and Coggeshall, 1983; Skinner et al., 1989; Jankowska, 1992; Flynn et al., 2011). They mediate information relayed between afferent and efferent fibers and, thus, aid in the interaction and integration of different spinal circuits. PN are highly versatile as their function depends on all the spinal circuits with which they are integrated, like motor function associated with central pattern generators (CPG; Ballion et al., 2001; Jordan and Schmidt, 2002) or with circuits relaying painful stimuli to supraspinal centers (Szentagothai, 1964). Thus, PN plays critical roles in transmitting information pertaining to motor, sensory and autonomic function (Jankowska, 1992; Conta and Stelzner, 2004). While we present evidence on the role of PN plasticity for motor recovery after spinal cord injury (SCI), herein we review factors influencing autonomic dysfunction and the contribution of PN plasticity towards this pathophysiology; and how such maladaptive plasticity might be targeted.

## PN Role in Motor Control After Spinal Cord Injury

PN exhibit a high level of neuroplasticity in humans with complete high cervical (C) SCI, in which transcutaneous electrical stimulation of lower limb nerves evoke motor responses in distal forearms (Calancie, 1991). Since such interlimb responses are absent in uninjured or partially injured SCI individuals, and there is a time-dependent decrease in the latency of upper limb muscle responses to electrical stimulation of peripheral nerves in the lower limb, this signifies an increase in regenerative sprouting below the injury to strengthen pre-existing synapses (Calancie et al., 1996). Electrophysiological recordings in cats with lesions at cervical spinal levels C5–C6 show that PN in the C3-C4 region integrate with the severed supraspinal tracts such as corticospinal, rubrospinal, reticulospinal and tectospinal tracts and relay information to distal motor neurons below the injury (Illert et al., 1977). PN mediate both excitatory and inhibitory postsynaptic potentials from corticospinal tracts to distal motor neurons after their transection at C5/C6 in cats (Alstermark et al., 1984). Primate studies have shown that following unilateral corticospinal tract transection in which C3-C4 PN remains intact, the lost grasping reflex is restored within 15 days (Sasaki et al., 2004; Alstermark et al., 2011). This indicates that PN contributes to voluntary motor function *via* disynaptic or polysynaptic pathways constituting corticospinal or reticulospinal tracts that are relayed *via* PN onto motor neurons. Similarly, a trisynaptic cortico-reticulospinal pathway has been reported in rats (Alstermark and Pettersson, 2014), wherein grasping reflex is controlled by both cortico-reticulospinal tract and polysynaptic connections in the spinal cord.

The CPG circuits are responsible for controlling rhythmic motor functions like walking, swimming, crawling, respiration, etc. As observed in isolated spinal cord preparations from neonatal rats subjected to chemical and electrical stimulation, the locomotor CPG is reported to modulate inter-limb coordination and stepping reflex mediated by PN, such that fictive motor responses occurred in phase opposition, similar to walking gait in adult rats (Ballion et al., 2001; Juvin et al., 2005; Zaporozhets et al., 2006). PN project both short and long tracts and their functions vary depending on their location, axonal length, and the direction of the signal relay. Tract tracing studies in uninjured rats using cholera toxin beta injections at L1/L2 in the ventral horn have documented the distribution of short-range PN both ipsilateral and contralateral within the lumbar enlargement (Liu et al., 2010). Similarly, labeling of neurons in the L2-L4 dorsal horns of rats using Phaseolus vulgaris leucoagglutinin and biotinylated dextran (BDA) tracers to differentiate between lateral and medial axonal projections show that the lateral fibers project along the entire length of the spinal cord whereas the medial fibers have shorter projections (Petkó and Antal, 2000). Long-range PN can be classified based on the direction of their axonal projections as ascending or descending, and long descending tracts create links between cervical and lumbar circuits involved in locomotor coordination (Brockett et al., 2013). Moreover, following mid-thoracic dorsal hemisection in rats, new circuits are formed between corticospinal tract axons and PN in the cervical spinal cord whose long tracts eventually connect with lumbar motor neurons (Bareyre et al., 2004).

Short-range PN are reported to be involved in forelimb grasping reflex and hindlimb motor coordination, among other functions, depending on their location in cervical vs. lumbar enlargements (Kostyuk et al., 1971; Alstermark and Kümmel, 1986; Gerasimenko et al., 2002). PN have an innate ability for *de novo* sprouting across the lesion in a feline midsagittal spinal transection model, despite their proximity to the axonal inhibitory protein, chondroitin sulfate proteoglycan (Fenrich et al., 2007; Fenrich and Rose, 2009). In addition to laminae VI and VII, lamina X of upper lumbar (L1/L2) spinal gray matter is considered as one of the presumptive sites for hindlimb CPG interneurons (Kjaerulff and Kiehn, 1996; Magnuson et al., 2005; Beaumont et al., 2006; Reed et al., 2006), and therapeutic preservation of such PN after upper lumbar contusion SCI is correlated with improved locomotor recovery (Patel et al., 2012). Application of N-methyl-D-aspartate (NMDA) between two opposite staggered thoracic spinal cord hemisections abolishes restored spontaneous hind limb functional recovery, suggesting ablation of sprouting PN prevents the formation of newly formed detour circuits after SCI; but the precise contribution of PN to functional recovery has yet to be characterized (Courtine et al., 2008). Using this injury model, Fouad et al. (2010) showed that constitutive activity of serotonergic (5-HT2c) receptors is required to elicit both spasticity as well as spontaneous locomotion *via* PN plasticity.

Injured PN axons respond positively to the presence of specific growth factors in mice with spinal hemisection (Anderson et al., 2018) and, therefore, PN plasticity is being targeted for its potential to elicit motor recovery after SCI. Accordingly, viral vectors have been used to label specific PN to delineate their roles in forelimb and hindlimb motor circuits in both naïve and mid-thoracic (T9) spinal contused rats (Sheikh et al., 2018). Specifically, highly efficient retrograde gene-transfer (HiRet) lentiviral Tet-On inducible expression vectors have been used to selectively label PN from specific locomotor circuits at C6-T1 or L1-L4. Notably, following T10 contusion SCI, there is a reduction in PN labeling in the T7 spinal cord compared to C3-C4 levels. Such viral vectors may be applied similarly to selectively target PN to activate or silence them to unveil their roles in motor recovery.

## Autonomic Dysfunction After Spinal Cord Injury

### Loss of Supraspinal Control

**Figure 1:**
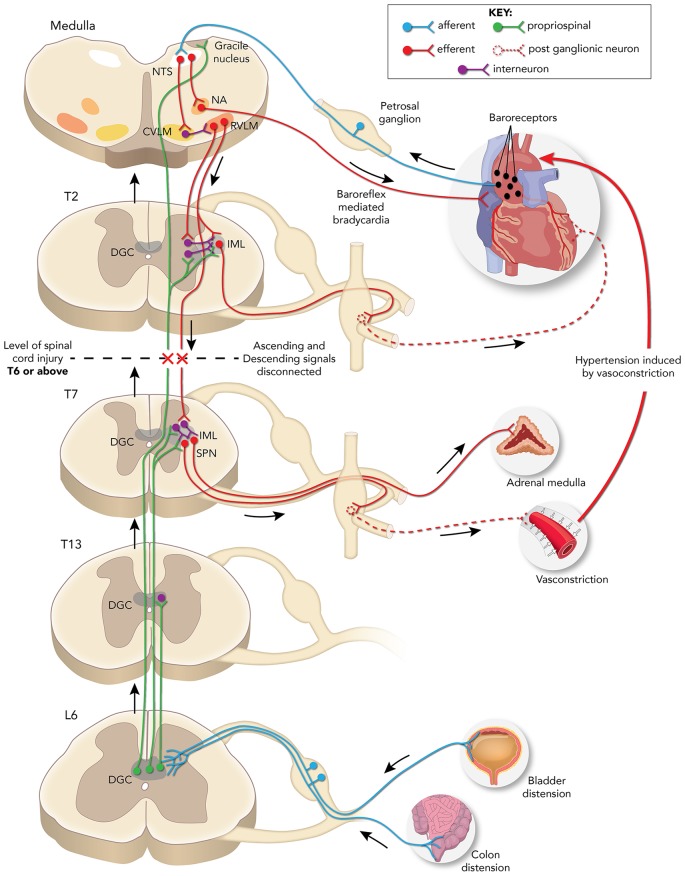
Schematic representation of neuronal pathways disrupted/rerouted by complete spinal cord injury (SCI) above the sixth thoracic (T6) spinal level associated with the development of autonomic dysreflexia (AD) evoked by noxious pelvic visceral distension. Sensory afferent fibers (blue) from the distended bladder or colon transmit noxious stimuli to short and/or long projection propriospinal neurons (PN; green) present in the dorsal gray commissure (DGC) at corresponding spinal levels. These PN then relay the signal rostrally to activate sympathetic preganglionic neurons (SPN) present in the thoracolumbar intermediolateral cell column (IML) directly or *via* interneurons to trigger adrenal hyperactivity, peripheral vasoconstriction, and consequent hypertension. The change in pressure is sensed by baroreceptors (black dots) in the aortic arch which relay the information *via* the nucleus tractus solitarius (NTS) in the medulla to the nucleus ambiguous (NA) that elicits: (1) a parasympathetic bradycardic response and/or; (2) concomitant neuromodulation *via* caudal and rostral ventrolateral medulla (CVLM and RVLM) projections to the IML directly or *via* interneurons to maintain normal blood pressure. After T6 SCI, the decentralized SPN elicits uninhibited vasoconstriction and hypertension, while the NA signals baroreflex mediated bradycardia. The lack of supraspinal regulation of SPN below the injury site maintains the hypertensive response until the noxious stimuli are removed, and maladaptive plasticity of both primary afferent fibers and PN are associated with the development of AD.

Profound autonomic dysfunction occurs after an SCI interrupts bulbospinal projections to sympathetic preganglionic neurons (SPN) in the thoracolumbar intermediolateral cell column (IML), which results in the loss of sympathetic modulation from the caudal and rostral ventrolateral medulla (CVLM and RVLM; Finestone and Teasell, 1993). Typically, the higher and more complete the injury, the direr the autonomic consequences as disconnection of SPN from bulbospinal neurons affects the regulation of sympathetic cardiovascular responses (Lehmann et al., 1987). Autonomic dysreflexia (AD) is an often debilitating condition characterized by erratic episodes of severe hypertension associated with or without bradycardia that occurs in patients with complete or incomplete SCI above T5/T6 levels (Karlsson, 1999). The incidence rate of this condition ranges between 20% and 70% in patients suffering from chronic SCI (Snow et al., 1978; Braddom and Rocco, 1991), and symptoms of AD include severe headaches, facial flushing, sweating, shivering, anxiety, piloerection, nausea, changes in vision, nasal congestion, et cetera (Kewalramani, 1980). Patients may also experience arrhythmias, atrial fibrillation, and paroxysmal hypertension (Ekland et al., 2008; Lee and Joo, 2017). Distension of pelvic viscera due to impacted bowel or full bladder is the most common trigger for AD, though other triggers can include ingrown nails and pressure sores (Snow et al., 1978; Harati, 1997; Krassioukov et al., 2003). Consequently, a massive increase in afferent signals reach the spinal cord and trigger a strong sympathetic response resulting in vasoconstriction below the injury (Figure 1).

Normally, supraspinal sympathetic regulation starts at the medullary neurons within the RVLM which project onto the SPN (Moon et al., 2002; Hou et al., 2013a). Axons of the SPN depolarize postganglionic neurons within prevertebral sympathetic ganglia that innervate target organs that are countered by the parasympathetic innervation by the vagus nerve (Strack et al., 1988). Baroreflex controls blood pressure *via* baroreceptors present in the heart and blood vessels that monitor and relay the rise/fall in pressure to the nucleus tractus solitarius (NTS) in the brainstem that then modulates the sympathetic response, accordingly. Supraspinal denervation results in the sudden depletion of descending excitatory signals resulting in a 50–70% loss of innervation to SPN (Llewellyn-Smith and Weaver, 2001). This, in turn, strengthens the existing spinal excitatory synapses and promotes reorganization of spinal circuits (Krassioukov and Weaver, 1996; Cassam et al., 1997). Notably, the severity of experimental AD in mice is correlated with the level of serotonergic inputs in the spinal cord that innervate the IML (Cormier et al., 2010), and grafting of neural stem cells derived from embryonic brainstem into T4 spinal transection sites in rats reduced the severity of experimentally induced AD (Hou et al., 2013b). These findings indicate that improving serotonergic connections between the brainstem and regions of IML below the lesion may aid in alleviating severity of AD. However, whether serotonergic innervation improves PN function or modulates SPN directly is uncertain.

Importantly, AD events are accompanied by adrenal hyperactivity with the level of adrenaline and noradrenaline increasing after SCI in humans and rodents that exhibit AD (Chiou-Tan et al., 1998; Leman et al., 2000; Teasell et al., 2000; Karlsson, 2006). SPN that innervate the adrenal gland show increased expression in the immediate early gene c-Fos after experimentally induced AD, a marker for cellular activity (Leman and Sequeira, 2002). It is posited that the alpha-adrenoceptors in blood vessels become hyper-responsive due to reduced presynaptic noradrenaline re-uptake or as a consequence of sympathetic dysfunction (Teasell et al., 2000). This hypersensitivity may also occur as a response to sympathetic mediated vasoconstriction in cutaneous blood vessels (Stjernberg, 1986). While adrenergic hypersensitivity is a noted contributing factor peripherally, we focus on the role of intraspinal plasticity in AD development.

### Synaptic Reorganization of SPN

The SPN neurons in the IML responsible for sympathetic activity extending from T1 to L2 spinal segments and SCI-induced changes in SPN phenotype are thought to influence the development of AD (Pyner and Coote, 1994; Krassioukov and Weaver, 1995b). SPN located in the IML sends sympathetic signals to the postganglionic neurons, which in turn relay the information to the target organs like heart, blood vessels, kidney, adrenal gland, etc. (Figure 1). In humans with complete SCI, the somal size of the SPN distal to the injury is reduced by almost half at 2 weeks after injury, but similar to normal sizes at longer time points (years; Krassioukov et al., 1999). Following high thoracic (T3) spinal cord transection in rats, dendritic morphologies of SPN show severe atrophy by the first week below the injury (Weaver et al., 1997; Klimaschewski, 2001) with cells closer to the transection site more severely affected than those more distal. While SPN recovers regular morphology after 2 weeks, they also show increased sensitivity to peripheral and visceral stimuli (Krenz and Weaver, 1998a). The increased expression of growth-associated proteins like GAP-43 and synaptophysin (pre-synaptic markers) distal to a T4-T5 transection site a week after injury in rats infers that new intraspinal circuits form in the IML after transection (Krassioukov and Weaver, 1996; Weaver et al., 1997). Such reorganization alters the nature of the input to SPN as noted by Llewellyn-Smith and Weaver (2001), who showed that glutamatergic inputs to SPN decreases whereas GABAergic inputs increase during this 2-weeks reorganization period.

### Maladaptive Plasticity of Nociceptive Afferents

Whether PN plasticity can be harnessed to improve both motor function while abrogating the development of AD is uncertain, based primarily on the overwhelming evidence that maladaptive neuroplasticity after SCI contributes to several pathological sequelae such as AD, cardiac arrhythmias, neuropathic pain, spasticity, bowel, bladder and sexual dysfunction (Collins et al., 2006; Mathias, 2006; Nout et al., 2006; de Groat and Yoshimura, 2006). In various animal models, both corticospinal and intraspinal circuits are reorganized following SCI (Tandon et al., 2009; Ghosh et al., 2010; Asboth et al., 2018), and such sudden increases in neuronal sprouting without suitable axon guidance cues and supraspinal modulation leads to an imbalance of inhibitory and excitatory signaling (see Brown and Weaver, 2012).

Complete T4 SCI in rats elicits afferent fiber sprouting into lower spinal levels that persist chronically (Krassioukov and Weaver, 1995a). Afferent fibers include Aβ, Aδ, and C-fibers, with calcitonin gene-related peptide (CGRP) expressed in all three types (McCarthy and Lawson, 1990; Lawson et al., 1993, 1996; Krenz and Weaver, 1998b; Wong et al., 2000; Marsh and Weaver, 2004). Maladaptive plasticity mediating pain and autonomic dysfunction has been attributed to an increased intraspinal sprouting of both CGRP immunoreactive nociceptive afferent C-fibers into the dorsal horns below the site of SCI (Christensen and Hulsebosch, 1997; Krenz et al., 1999), as well as increased serotonergic fiber densities rostral to the injury (Oatway et al., 2005). Altered glutamatergic signaling after SCI contributes to abnormally increased activity in spinal sympathetic reflex circuits (Maiorov et al., 1997), and Krenz and Weaver (1998b) reported that hyperreflexia observed after injury was due to due sprouting of unmyelinated C-fibers to increase afferent fiber input onto interneurons. While the primary contributing factors for the development of AD after injury are thought to be maladaptive sprouting of afferent fibers and PN, their individual contributions to this syndrome remain uncertain.

For example, the prevention of C-fiber sprouting using intrathecal nerve growth factor (NGF) neutralizing antibody or trkA-IgG fusion protein in T4 spinal transected rats decreases CGRP-fiber density in association with reduced AD symptoms in experimental rats undergoing experimental colorectal distension (CRD; Christensen and Hulsebosch, 1997; Krenz et al., 1999; Marsh et al., 2002; Weaver et al., 2006). Moreover, complete T4 transection significantly increases sprouting of C-fibers innervating the distal colon into the lumbosacral spinal cord (Hou et al., 2009), and over-expression of NGF using viral vectors injected into the lumbosacral dorsal horn increases the severity of AD in response to CRD (Cameron et al., 2006). On the contrary, while viral over-expression of semaphorin 3a, a chemorepulsive factor for C-fibers, reduces the severity of CRD induced hypertension in parallel to reduced sprouting of nociceptive visceral afferent C-fibers into the spinal cord, it does not completely abolish the pathology. Alternatively, increased CGRP+ fiber sprouting following complete T4 spinal transection does not accompany the development of AD in some strains of mice (Jacob et al., 2003), which points to an underappreciated role played by PN plasticity in manifesting AD.

## Autonomic PN

Autonomic PN are pre-sympathetic as they innervate the SPN to control their level of excitation (Gebber and McCall, 1976), but the role of PN in eliciting sympathetic control is highly underappreciated. Supraspinal centers play critical roles in sympathetic regulation when compared to interneurons, but when this control is lost due to pathological conditions like SCI, then the PN take over sympathetic regulation by directly acting on the SPN to evoke responses (Schramm, 2006). The primary afferent fibers that carry the signal to the spinal cord are not directly linked to the SPN, they communicate with the SPN primarily *via* interneurons that may be excitatory or inhibitory in nature depending on the type and location of the stimuli (Chau et al., 2000). PN with sympathetic function predominantly present in the dorsal horn of cats were identified electrophysiologically by Gebber and McCall (1976) by cross-correlating PN spikes with signals in sympathetic nerves, and all signals with low inter-spike intervals (<20) were inferred to be from sympathetic PN.

BDA anterograde tracer injected into the central gray matter at rat L6-S2 spinal levels shows that ascending PN extend projections up to cervical levels through lamina X, and retrograde labeling using wheat germ agglutinin-horseradish peroxidase show projections to laminae VII and VIII at lumbar and thoracic levels indicating the anatomical locations of ascending PN (Matsushita, 1998; Petkó and Antal, 2000). Similarly, when Phaseolus vulgaris leucoagglutinin tracer is injected in lamina X of the lumbosacral cord, PN projection axons in the dorsal column and ventrolateral funiculus at the cervical level are labeled (Wang et al., 1999). Transynaptic retrograde tracing with pseudorabies virus injected into the adrenal gland, kidney, and stellate ganglion label PN in lamina VII and X extending from T4–T13, T11–T13 and C1–C4 spinal levels, respectively (Strack et al., 1989; Jansen et al., 1995; Tang et al., 2004). Moreover, a population of GABAergic interneurons innervating SPN has also been identified in the spinal central autonomic area (Deuchars et al., 2005), also termed the dorsal gray commissure (DGC, lamina X). Importantly, bladder distension in rats with spinal transection activates afferent fibers to increase the number of c-Fos-immunoreactive neurons in the DGC/lamina X and lateral dorsal horn of the L6/S1 segments (Vizzard, 2000), indicating that lumbosacral PN disconnected from supraspinal control are activated *de novo* by visceral stimuli following SCI.

### Plasticity of Autonomic PN After SCI

Ascending PN plasticity is believed to contribute to the development of AD. These neurons relay visceral sensory information towards the SPN in the thoracic cord (Rabchevsky, 2006; Schramm, 2006). Functional plasticity of PN comprising spinal sympathetic circuits after SCI in the rat was reported by Krassioukov et al. (2002) investigating sympathetically-correlated interneuron responses to applied stimuli below the injury hours (acute) or 1 month (chronic) following T3 transection. It was found that only in the chronic stage of injury, interneurons’ electrical activities were cross-correlated with renal sympathetic nerve activities during CRD and skin pinching caudal to the injury. This study showed that plasticity occurs within somatosensory PN that modulate sympathetic activity in the weeks after injury and that peripheral stimulation in chronic SCI rats activates more interneurons compared to acute SCI animals.

Accordingly, BDA tracers injected into the lumbosacral cord following T4 transection show more labeling of ascending lumbosacral PN fibers that originate at the DGC and terminate proximal to Fluorogold-labeled thoracic SPN (Hou et al., 2008). The visceral sensory afferent fibers terminate at the DGC and the signal is relayed up towards the target regions *via* intraspinal projection neurons (Pascual et al., 1993; Hosoya et al., 1994; Al-Chaer et al., 1996; Matsushita, 1998; Vizzard et al., 2000). Extended CRD trials performed 2 weeks after T4 SCI increase neuronal activity throughout the lumbosacral DGC, as shown by conspicuously higher numbers of c-Fos positive neurons in comparison to sham controls (Hou et al., 2008). However, it is currently unclear whether ascending DGC neurons modulate SPN activity by directly projecting to the SPN or indirectly *via* interneurons. After SCI, the supraspinal control is lost but the SPN are activated by peripheral afferent stimuli whose signal is relayed *via* ascending PN to trigger a sympathetic response, resulting in hypertension (Figure 1; Schramm, 2006).

Excitatory interneuron connectivity with SPN preferentially increases after T3 spinal transection in mice, and repeated CRD induces an increase in these excitatory interneurons co-labeled with vGlut2 (Ueno et al., 2016). This indicates that visceral noxious stimuli trigger excitatory PN sprouting that augments SPN signaling leading to AD. The existence of PN relaying afferent input to SPN may be sufficient to trigger sympathetic responses, and the lack of supraspinal modulatory signals results in uninhibited continuous sympathetic firing leading to hypertensive crises (Rabchevsky, 2006). Thus, plasticity of ascending PN and local sensory afferent fibers augment the noxious input to activate SPN, which in turn triggers a volley of sympathetic discharge and consequent vasoconstriction leading to AD (Figure 1).

### Techniques for Targeting PN Selectively

Electrophysiology and retrograde tract-tracing are the most common tools used to identify the tracts involved in autonomic function. Although several studies labeling long descending PN and short tract PN exist, it is still highly challenging to specifically target and label ascending propriospinal tracts after injury due to unintended labeling of fibers en passage. Moreover, it is reported that transsynaptic labeling by pseudorabies virus is significantly reduced in the T4 transected spinal cord (Duale et al., 2009), thus limiting the availability of effective tools for tracing the course of these neurons after injury. While selective inhibition of interneurons may aid in understanding and possibly alleviating the development and/or symptoms of AD, silencing interneurons involved in eliciting AD without affecting the interneurons involved in other important functions adds to the complexity when no effective tools exist to specifically identify interneurons. Using contemporary chemogenetic tools designed to silence specific neuronal populations of PN, it may now be possible to target and delineate the role of PN plasticity in the development of AD.

In this regard, Kinoshita et al. (2012) reversibly silenced PN involved in primate forelimb control by infecting the ventral horn of C6-T1 spinal segments in macaque monkeys with HiRet lentiviral vectors that contained enhanced tetanus neurotoxin toxin light chain (eTeNT) and eGFP coding sequences downstream of the tetracycline-responsive element (TRE) sequence. An adeno-associated viral vector containing the Tet-On sequence (AAV-Tet-On) was injected in the intermediate zone of cervical C2-C5 levels prospectively containing the cell soma, and only cells that had been transfected by both vectors were silenced by the Tet-ON reaction with doxycycline. Since PNs extend through these areas, they were predominantly labeled and selectively silenced by these vectors, thus enabling selective and temporal labeling with GFP and simultaneous silencing of the forelimb locomotor circuit encompassing the motor cortex, PN and motor neurons controlling hand movement. In theory, a similar modality may be used to identify the role of PN in rodent SCI models that elicit AD by selectively silencing lumbosacral PN that relay signals from afferent fibers below the lesion to SPN during noxious CRD. Specifically, the GFP tagged Hiret lentiviral vectors can be used to retrogradely label ascending lumbosacral PN innervating the thoracic IML, and by injecting AAV-TetON vectors to transduce PN in the lumbosacral DGC, it would enable selective targeting and silencing of the dual-labeled ascending PN (Eldahan and Rabchevsky, 2018).

## Conclusion

PN plasticity after traumatic SCI plays a critical role in improving not only spontaneous motor function but also potentially aggravating autonomic dysfunction. In addition to afferent C-fiber sprouting and loss of supraspinal regulation of the IML, maladaptive plasticity of PN below the level of high thoracic SCI appears essential for manifesting chronic autonomic dysfunction. Thus, while selectively promoting PN sprouting might serve as a promising therapeutic target for specific motor functional recovery, it may also be prevented selectively to combat AD development and/or severity, and the advancement of strategies to reversibly silence these interneurons will help further our understanding on their role in AD.

## Author Contributions

FM wrote the first draft of the manuscript. SP reviewed the manuscript. AR critically evaluated the manuscript and wrote the final revised version. All authors contributing to the manuscript have read and approved the submitted version.

## Conflict of Interest

The authors declare that the research was conducted in the absence of any commercial or financial relationships that could be construed as a potential conflict of interest.
